# Humanity’s Attitudes about Democracy and Political Leaders: Patterns and Trends

**DOI:** 10.1093/poq/nfab056

**Published:** 2021-11-26

**Authors:** Christopher J Anderson, Damien Bol, Aurelia Ananda

## Abstract

For decades, researchers have examined people’s beliefs across countries and over time using national samples of citizens. Yet, in an era when economies, societies, and policymaking have become increasingly interconnected, nation-states may no longer be the only or most relevant units of analysis for studying public opinion. To examine what people think about politics on a global scale, we develop tools for measuring public opinion that allow us to transcend national and regional boundaries. Starting with the world as the unit of analysis and humans as the relevant population, we measure and then explore patterns and trends in human preferences for democratic government and political leaders with the help of surveys collected around the world since 1994.

Scholars of comparative and international politics have long been interested in ordinary citizens. Using data collected by national and international survey projects, researchers for many decades have examined differences in people’s opinions within and across countries as well as over time in order to understand what citizens think and want. While, for virtually all of modern political history, the most prominent populations of interest have been those of nation-states, we have come to live in a world where politics and policies are made on a global scale or have global implications—policies to fight climate change or accommodate migration come to mind. As a consequence, it has become increasingly important for political decision makers to understand what people the world over think about the issues of the day.

For example, when deciding whether to take measures to protect human rights, safeguard free and fair elections, intervene in civil conflicts, or advance gender equality, policymakers at global institutions like the UN, WTO, or World Bank may wish to know whether nonelites the world over support democracy or how they view the role of women in society. As a result, the global population’s preferences may have the power to act as a constraint on elite decisions by establishing a zone of acquiescence and limits to what is acceptable worldwide. Global opinion may matter to voters as well—there is growing evidence that they judge their own country’s institutions and outcomes with an eye toward outside benchmarks (Kayser and Peress 2012; de Vries et al. 2021). One such benchmark may be how people the world over think about key issues of our time.

Thus, we argue that the traditional approach toward public opinion for understanding politics butts up against politics in a globalized world. That is, because the nation-state may no longer be the only or most relevant unit of analysis for understanding what or how people think, it has become increasingly important to develop an understanding of what people globally and as a species think about the key issues of our time. Do humans—rather than Belgians, Germans, or Indonesians—actually like democracy, or do they prefer having a strong leader who does not have to bother with democratic processes? Do they think men make better political leaders than women? Equally importantly, how much global disagreement is there about such questions and have these preferences evolved over time?

While these questions sound simple, existing research designs and available data sources are not readily equipped to answer them with precision because of imperfect, limited, and uneven sampling of people and countries around the world. Below, we therefore start with the world as the unit of analysis and humans as the relevant population in order to develop a novel way of analyzing existing surveys to gauge public opinion on a global scale. Using data from the World Values Survey (WVS) and other sources, we develop an original estimation technique similar to Multilevel Regression with Poststratification (MRP), which has been used to estimate public opinion in subnational units like regions or states when the survey sample is national and not representative of populations at the subnational level. We apply this framework to estimate public opinion globally and conduct a series of tests to validate our new measures of human preferences for democracy and types of political leaders. While aimed at measuring public opinion on a global level, our approach does not assume that people’s views on these issues are homogeneous, nor that country-specific variables are irrelevant as determinants of opinions. Quite the contrary: a key element of our analysis is that we use information about countries’ structural conditions and population characteristics to predict global public opinion.

Our analysis shows that, over the past quarter century, humanity consistently has had a strong preference for democratic government. There is little disagreement over its rightfulness, regardless of world region. However, while the baseline of support for undemocratic leadership is low, too, the average human has become more comfortable with leaders not subject to the usual democratic constraints and continues to express a preference for male over female leaders. Strikingly, the evolution of these two attitudes varies by region of the world. Although they have been quite stable in the West over the last decade, they have increased substantially in Asia and Africa since the mid-2000s. Finally, we find that humanity’s preferences are shaped less by individual differences than by geography: there is substantial geographic heterogeneity in humanity’s taste for strong leaders and the idea that “men make better leaders than women,” depending on world region. In contrast, variations in preferences for democracy and types of political leaders arising from demographic characteristics are much smaller, with differences among age groups or genders outweighed by differences due to where individuals live.

We begin by placing our question in the broader context of global political developments and the potential relevance of understanding global opinions. We then develop a new way to estimate global public opinion and the average human response to a variety of questions about democracy and political leaders. Next, we present and discuss the results of these responses for humanity as a whole, as well as for subgroups of the human population, by world region and demographics. A final section concludes.

## Going Global

How people think about and engage with politics are long-standing questions in political science. Over the past three centuries, the role of “the public” in political life has undergone a dramatic evolution around the world, with members of the public ceasing to be mere subjects and transforming into participants with the agency to be legitimate critics of the state (Luhmann 1997, 2018). Because politics most commonly has been conceptualized as the politics of nation-states since the emergence of the international state system, students of politics primarily focused on members of the national community as the population of interest. As a result, understanding and analyzing the populations of territorially defined units (typically nation-states) became the essential building block of research on political behavior and public opinion.

While this remained the case for much of the twentieth and into the twenty-first century, trends toward regional integration, regionalized world politics, and globalization have begun to create a recognizable politics beyond the nation-state. Thus, scholars have begun to ask whether there is, for example, a European Demos or entertain the idea that a global public has been emerging (Habermas 1991). Yet exactly what such a Demos might look like or how we would detect and understand it is far from clear, and there is a lively debate over the feasibility and desirability of a global body politic (List and Koenig-Archibugi 2010).

Ideas about a global opinion climate or Zeitgeist lay bare questions of what the relevant population of interest should be and, importantly, how it might behave. In a world where supranational, transnational, and global governance mechanisms and institutions have been developing for many years, where pressing policy problems traverse national boundaries with ease, and where communication networks allow citizens to be increasingly connected without regard to the limits imposed by the nation-state, one logical next step would be to measure the opinions of all people on the planet rather than exclusively the members of specific (territorial) political communities.

Thus, our modest aspiration is to find ways of measuring all people’s views on matters important for understanding the human experience, that involve politics beyond the nation-state, or that can be thought of as representing a global public view. Thus, as a purely empirical matter, we want to know what humans globally think about key matters of governance, not because these preferences are globalized in some traceable way at the level of individuals but because policies and politics are, and because we do not know to what extent humans actually agree on basic aspects of governance. Seen from the perspective of global public opinion, then, this means establishing a worldwide baseline regarding the level and variation in humanity’s opinions about politics.

To do so, we focus on people’s views about democracy and political leaders, and for several reasons. As a practical matter, questions about democracy and political leaders have been asked across most cross-national survey projects like the World Values Survey (WVS) conducted to date. As such, they provide the necessary database for developing and testing our new method for devising a useful blueprint for studying people’s views on a global scale. On conceptual and empirical grounds, questions about people’s preferences for democracy and equality continue to be of global importance. The spread of democracy and message of its relative success have increased familiarity with the concept, penetrating even the most closed societies (Kirsch and Welzel 2019). However, while it seemed that democracy had “won” the war of ideologies in the aftermath of the Cold War, there has been much debate about democratic “backsliding” or “democratic recession” in recent years, with electoral dictatorship either becoming more popular or replacing democratic rule outright (Diamond 2015; Bermeo 2016; Levitsky and Ziblatt 2018; Brunkert, Kruse, and Welzel 2019). One related empirical question for students of public opinion is whether there is evidence that humanity currently is or has become less enamored of democracy and democratic institutions.

Relatedly, the emergence of populist leaders and global trends toward greater gender equality raise important questions about equality, human rights, and the kinds of political leaders that people prefer. Thus, we also investigate whether support for democracy as a form of government coexists with preferences for particular types of political leadership. There is reason to assume that people’s preferences for democracy are consistent with political values that Inglehart and collaborators have termed “emancipative” (Inglehart and Norris 2003; Inglehart and Welzel 2005). Thus, on one hand, valuing democracy should be connected to preferences for equality—for example, for all adults, rather than just men, to have access to political leadership positions. If this is the case, the preference for democracy should be incompatible with a strong preference for male leaders, for instance.

At the same time, while people generally say they like democracy, they have different and sometimes inconsistent ideas about what it means (Kirsch and Welzel 2019; Kruse, Ravlik, and Welzel 2019). Thus, citizens commonly perceive democracy as working poorly because it can seem chaotic and disorderly (Hibbing and Theiss-Morse 2002)—a “flaw” that people may believe can be “fixed” with the help of leaders who are seen as “strong.” Unsurprisingly, too, political leadership traits are gendered, such that maleness is often associated with strength, order, and competency, especially in times of uncertainty (Huddy and Terkildsen 1993; Holman, Merolla, and Zechmeister 2016). As a result, a preference for democracy could feasibly coexist with a preference for male leaders. Thus, to measure people’s preferences for political leadership, we also examine support for so-called “strong leaders.”^1^

## Analyzing What (All) People Want

The global diffusion of survey research and the emergence of numerous collaborative cross-national survey projects in recent decades have allowed researchers to study political behavior in many countries around the world.^2^ We aim to build on these efforts and take the next logical step. We are richer in global survey data than we have ever been in the history of humankind. However, because we are not quite rich enough to rely on surveys alone and are unlikely to be so for some time to come, we build on an existing set of extensive but incomplete surveys that have broad geographic coverage to produce a more complete picture of what humans think and want. In particular, our estimation of what humanity thinks about democracy and political leaders relies on the “All-Rounds-Country-Pooled Dataset” from the WVS, the largest set of cross-country surveys ever assembled (Inglehart et al. 2020).

The WVS has an important limitation: despite the name, it does not cover all countries in the world, it does not sample world regions evenly, and it does not cover the same countries across all years. Thus, even with a greatly expanded set of cross-nationally comparative surveys, the sampling of human respondents is imperfect. While many researchers have ignored these limitations and analyzed whatever data are available for any set of countries in any survey wave, some have taken this limitation seriously. The most common approach is to fill in the gaps between survey years using a smoothing technique that extrapolates a reasonable estimate for the missing time points (Stimson 1991). This method also allows researchers to combine several survey questions and datasets to create a latent measurement of public opinion over time at the national (Beck 1989) or subnational level (Caughey and Warshaw 2016).

This “mood” method has been successfully used (and expanded) by Claassen (2019) to study public support for democracy across time, and it has the advantage of maximizing the spatial and temporal coverage of the data by combining a maximum of observations. This “coverage maximization” is also at the heart of several projects that seek to merge data from a maximum of surveys worldwide (e.g., Neundorf et al. 2017; Klassen 2020). However, this approach requires researchers to combine survey questions that do not always have the same wording. Hence, the estimate is often not directly interpretable; instead, it produces a latent value that does not refer to a specific response scale.^3^ Another disadvantage of the “mood” method is that it cannot be easily used to impute the values to countries that have *never* been covered in any survey, a quite common occurrence. In the WVS, for instance, some larger countries like Yemen and Turkmenistan have not been surveyed since it launched in 1981.

Our approach is similar in spirit but goes beyond this method in three important ways. First, because we want to measure global opinion, we fill in *all* of the blanks: temporal gaps between survey years as well as geographical gaps between surveys never included in the WVS. Second, we impute values for single survey questions, which means that they are directly interpretable in view of the related response scale. Third, we take an individual-level approach that does not exclusively rely on countries’ (aggregate) averages. Instead, we capture the degree of agreement/disagreement between human beings, providing us with information about central tendencies and distributions of opinions. To achieve these goals, we use the WVS in tandem with the Quality of Government (QOG) dataset that provides a wide range of information for all countries and territories of the world since 1946. The “Time Series Standard Dataset” from the QOG project gathers information about countries’ political, economic, and social characteristics from multiple sources and datasets (e.g., the United Nations, the World Bank, etc.) into one (Teorell et al. 2021).

The survey questions we investigate below have two desirable properties: first, they address universal questions that have been studied for many years; second, they have been asked regularly over decades as part of the WVS and thus provide extensive coverage across many countries. These include support for a democratic political system, support for the idea that the political system should be governed by a strong leader who does not have to bother with parliament and elections, and agreement with the statement that men make better political leaders than women. These questions have been asked in about 250 surveys across countries and years of a total of more than 300,000 respondents (the question wording can be found in Appendix A). Finally, because the proportion of “don’t knows” is also relatively small (4 to 6 percent), we can safely exclude them to concentrate on expressed preferences without affecting the results too much.^4^

To ensure that the WVS covers a sufficient portion of the world’s population in order for us to draw inferences about the portions it overlooks, we calculate its global coverage in two ways. First, we examine coverage by comparing the number of countries included in the WVS (in each wave) with the official number of countries in the world (defined by membership in the UN). Second, we compare the WVS’s coverage of the world’s population (the total population of all the countries included in the WVS) with the world’s population according to the nongovernmental organization Worldometers.^5^

As table 1 shows, since the survey items we examine below first began to be included in 1994, the proportion of countries covered ranges from 18 to 29 percent. This relatively low level of coverage reflects the fact that most of the world’s smallest countries (e.g., Andorra, Luxembourg, Lesotho, etc.) have not been surveyed by the WVS. However, the survey’s coverage of the world’s population, rather than just the number of countries, is significantly greater, ranging from 30 to 74 percent per wave.

**Table 1.** Global coverage of the WVS questions

WVS wave	Period	Country coverage (%)	Population coverage (%)
Support for strong leader			
3	1994–1998	24	30
4	1999–2004	19	47
5	2005–2009	26	55
6	2010–2014	26	52
7	2017–2020	23	58
Support for democracy			
3	1994–1998	25	48
4	1999–2004	21	69
5	2005–2009	28	74
6	2010–2014	29	71
7	2017–2020	23	58
Support for male leader			
3	1994–1998	26	69
4	1999–2004	22	69
5	2005–2009	28	74
6	2010–2014	23	71
7	2017–2020	18	58

## Analysis: Estimating What Humanity Thinks

Our analysis is similar in logic to simulations obtained via a method known as Multilevel Regression with Poststratification (MRP), originally designed to estimate public opinion at a subnational level with the help of data collected at the national level (Gelman and Little 1997; Leeman and Wasserfallen 2017). This method has since been used to gauge both local public opinion (Lax and Phillips 2009; Tausanovitch and Warshaw 2014) and local vote intention polls (Lauderdale et al. 2020). In a nutshell, our analysis proceeds in two steps. We first create a “humanity dataset”—a sampling frame in which each line represents 10,000 (adult) human beings.^6^ This dataset simulates a worldwide random sample of adults, each selected with a probability of one in 10,000. Conventionally, unstandardized weights are simply the inverse of the sampling fraction, so each “respondent” in the humanity dataset can be thought of as having an unstandardized design weight of 10,000. It is in this sense that each element of the humanity dataset “represents” 10,000 of their fellow citizens.^7^ In a second step, we estimate a multilevel regression predicting preferences for democracy and political leaders with the WVS dataset and use the results to impute responses for the synthetic respondents in the humanity dataset. Below, we briefly explain the logic behind these two steps (for further details, please see the Supplementary Material).

### Step 1: Creating a Humanity Dataset

In the first step, we generate synthetic respondents based on five key demographic characteristics: gender, education, age, urbanization, and income. However, instead of naively assuming these characteristics to be distributed randomly across all human beings, we use data from the WVS and the QOG to simulate the joint distributions of each of these for countries not covered in the WVS. Concretely, we first calculate their means and standard deviations at the country-year level and the intercorrelations among them in the WVS dataset. In total, this produces 20 parameters (five means, five standard deviations, and ten correlations). Then, we add those parameters to the QOG dataset, and estimate a series of OLS regressions of the following specification:

(1) $$Y_{c,t}=\;+{\;\delta Z}_{c,t}+{\gamma T}_{c}+{\varphi T}_{c}^{2}+e_{c,t}$$

In equation 1, *Y_c,t_* is the outcome variable. It represents each of the 20 parameters of the joint distributions of the five demographic covariates for country *c* in year *t*. ***Z*** is a vector of covariates of socioeconomic and political covariates available in the QOG that consists of variables likely to affect attitudes toward democracy and political leaders, including indicators of countries’ demographics (e.g., population size, age, religion), socioeconomic conditions (e.g., GDP, access to telephone landlines), and political conditions (e.g., corruption, democratic quality). The full list of these covariates including descriptive statistics are shown in Appendix B). Finally, equation 1 includes a series of polynomials capturing time trends ($T_{c}\;\mathrm{and}\;T_{c}$^*2*^, which respectively capture the year and year squared), and an error term ($e_{c,t}$).

We limit the number of covariates for two reasons. First, although the QOG dataset includes additional variables, many of them have missing values for small countries or do not cover each year during the 1994–2020 period we investigate. Second, because there is a trade-off between in-sample and out-of-sample validity, an over-fitted regression that uses too many covariates tends to be too specific to accurately predict the outcome value of new observations.^8^

We then use the results from these regressions to derive the predicted values of the parameters for all countries in the world and for all years for the period of interest (1994–2020), including those not covered by the WVS. Finally, in a new dataset, we generate synthetic random respondents and samples for each country so that their number is proportional to the country’s population; in attributing to them values for the five demographic variables we impose that, for each country-year, their joint distributions exactly fit the parameters predicted by the OLS regressions.^9^ In total, we thus have a dataset with millions of lines.

**Figure 1.**
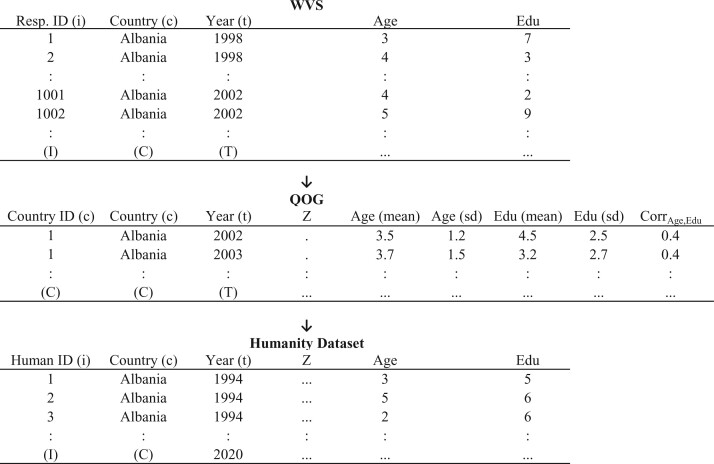
**Schematic representation of step 1.** Each row in the first matrix (top) represents a survey respondent in the WVS dataset from country *c* and year *t* and for whom we have measures on sociodemographic covariates like Age and Education. Each row in the second matrix (middle) represents a country-year for all countries *c* and years *t* in the QOG dataset. The arrow connecting the two indicates that parameters of the joint distributions of sociodemographic variables calculated on the basis of the WVS dataset are then appended to the QOG dataset. Each row in the third matrix (bottom) represents 10,000 synthetic human beings with sociodemographic characteristics (here, Age and Education) that exactly respect the predicted parameters of the joint distribution for each country *c* and year *t* between 1994 and 2020. The arrow between the second and third matrix indicates that the results of the regressions predicting the parameters of the joint distributions using the QOG covariates ***Z*** are used to simulate synthetic human beings in the Humanity dataset.

The matrices in figure 1 show this first step and how the datasets are connected with each other schematically. In the first dataset, we start with the aggregated WVS data that include all survey waves and all countries. We use this dataset to calculate the parameters of the joint distributions of the five sociodemographic covariates for each country and each wave (for the sake of simplicity, figure 1 includes only two parameters—Age and Education). We then add those parameters to the QOG dataset, as shown in the second matrix of figure 1. We subsequently estimate a regression predicting the parameters of the joint distribution of sociodemographics using the covariates ***Z*** from the QOG dataset and then use these results from the regression to impute the predicted parameters of all countries and years from 1994 to 2020, including those not covered by the WVS dataset. Finally, we create the humanity dataset (the third matrix) that has one line for 10,000 human beings and that contains sociodemographic variables that exactly respect the predicted parameters of the joint distribution of sociodemographics. The humanity dataset covers each year between 1994 and 2020.

An important validation of the accuracy of the humanity dataset includes a series of out-of-sample tests (reported in Supplementary Material part A). For these, we focus on the countries included in the WVS and for which we have both the covariates and outcome variables. We remove 45 country-years or 15 entire countries at random. These correspond to around 30 percent of the total number of observations, which is approximately the coverage of the WVS for our outcome variables (see table 1) and thus the scope of the imputation that we execute in our analysis. We then estimate the OLS regressions on the reduced sample and show that the predictions of the regressions allow us to recover the values of the randomly removed countries and country-years. Further, Part A of the Supplementary Material includes a qualitative assessment of the distributions of demographics in developing and developed countries. We observe that our humanity dataset fits the conventional wisdom—for example, that women have limited access to education in developing democracies. Taken together, the results all provide support for the quality of the humanity dataset, and especially the quality of the synthetic samples relative to those covered by the WVS.

### Step 2: Simulating What Humanity Thinks About Democracy and Political Leaders

Having created the humanity dataset, the second step of the analysis consists of simulating the missing responses of our synthetic humans to survey questions about democracy and political leaders. To do so, we use the WVS dataset to estimate a series of multilevel OLS regressions to predict responses with the following specification:

(2) $$Y_{i,c,t}=+\theta X_{i,c,t}{\;+\;\delta Z}_{c,t}+{\;\rho W}_{i,c,t}+{\gamma T}_{i,c}+{\varphi T}_{i,c}^{2}+e_{i,c,t}$$

*Y_i,c,t_* is the outcome variable. It represents responses to the three survey questions of interest for each survey respondent *i* in country *c* at year *t*. ***X*** is a vector capturing the five demographic covariates of the humanity dataset (sex, education, age, urbanization, and income). ***Z*** is a vector of covariates of socioeconomic and political covariates available in the QOG (see Appendix B).***W***is a vector of cross-level interactions. In order to not overfit the model, we did not include an interaction between all country-level and individual-level covariates. Instead, we focus on those that are more meaningful in the context of support for democracy and political leaders.^10^  $T_{c}\;\mathrm{and}\;T_{c}$^*2*^ capture time trends. $e_{i,c,t}$ is the error term clustered by country-year.^11^ We subsequently use the results from these regressions to simulate what would have been the answers of synthetic respondents in the humanity dataset covering all countries of the world between 1994 and 2020.

**Figure 2.**
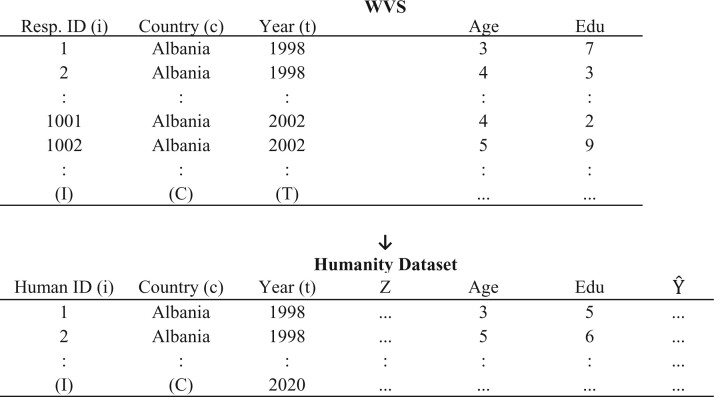
**Schematic representation of step 2.** Each row in the first matrix (top) represents a survey respondent from country *c* and year *t* in the WVS dataset and for whom we have measures on sociodemographic covariates like Age and Education, a democratic attitude *Y*, some QOG covariates ***Z***, and some cross-level interactions ***W***. Each row in the second matrix (bottom) represents 10,000 synthetic human beings with sociodemographic attributes (here, Age and Education) for each country *c* and year *t* between 1994 and 2020. The arrow connecting the two indicates that the result of the regression predicting Ŷ with sociodemographic covariates, (country)-level covariates ***Z***, and cross-country interactions ***W*** are used to predict the value of the outcome variable Ŷ in the Humanity dataset.

Figure  2 presents the second step schematically. In the WVS dataset, we estimate a regression predicting the outcome variable *Y* with the help of sociodemographic and QOG covariates ***Z*** that we previously appended to the WVS dataset. We then use the results of this regression to impute the predicted value of the outcome variable Ŷ for all synthetic human beings of the humanity dataset between 1994 and 2020. For this last step, we produce “multiple imputations,” a conventional method to impute missing values (King et al. 2001). Instead of using the predicted value of the regression as the imputed value, multiple imputations introduce a stochastic element in the prediction that has the distributional properties of the error term ($e_{i,c,t}).$ In turn, this procedure produces results that better reflect the uncertainty associated with real respondents answering real survey questions.^12^

Part B of the Supplementary Material includes a series of validation tests including out-of-sample tests (including randomly removing 75 country-years of the WVS dataset—that is, around 90,000 respondents, and 15 entire countries). In addition, we systematically compare our predictions for countries *not* covered in the WVS (that is, the responses of our synthetic respondents) with survey data about democracy from other sources available for these countries. The two are strikingly similar. Finally, we also show the difference between our estimates and the raw estimates from the WVS to show evidence of the usefulness of our approach. Ours give a more precise estimate of global public opinion than one that would consist of taking all WVS surveys available at a point in time and considering this to reflect the world population. This last approach is particularly problematic, as opinion averages change erratically from year to year depending on which countries are covered in the survey.

## Results

We present our substantive results in three steps. First, we inspect the overall distributions and trends in human preferences for democracy and political leaders by examining how positive or negative opinions are as well as how unanimous or spread out they are across the human population. Moreover, we examine whether these preferences have changed over time. Second, we investigate differences in opinions by demographic characteristics to see whether there are meaningful discrepancies as a function of people’s individual-level characteristics. Finally, we evaluate how much these preferences vary, depending on the region of the world people live in, and under democracy and dictatorship.

### What Humans Want and How Much They Agree

**Figure 3.**
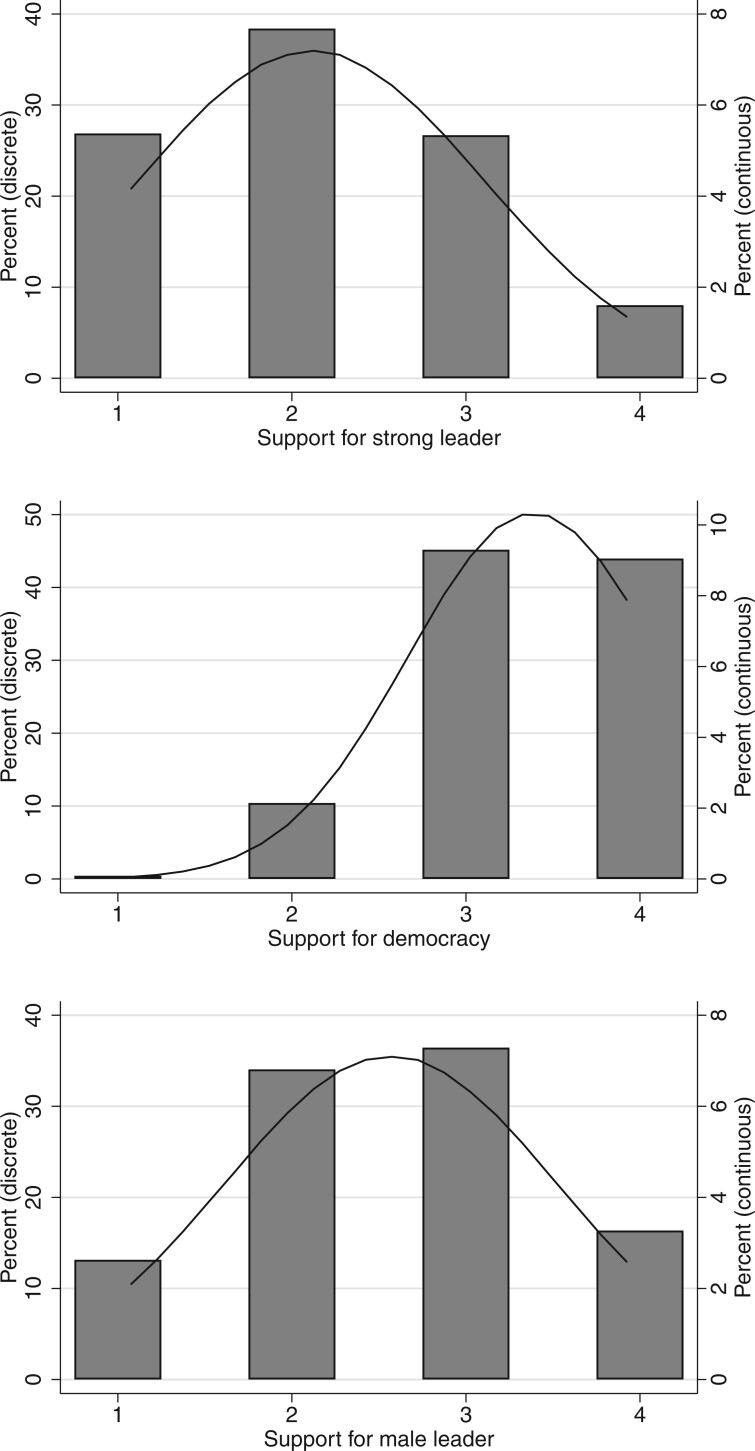
**Humanity’s preference for democracy and political leaders.** The imputation method gives continuous estimates. The solid lines show the distribution of these continuous estimates. Bars shows the proportion of human beings in each category of the outcome variable: (1) very bad, (2) fairly bad, (3) fairly good, or (4) very good when we round the continuous estimates to the closest discrete value.

Figure  3 shows the overall distributions of responses from the complete humanity dataset, along with their distribution bucketed on the four-point scales. The overall means indicate that humanity is very much in favor of democracy (3.39; sd = 0.72), believes men make better political leaders than women (2.57; sd = 0.95),^13^ and is skeptical about having a strong leader who does not have to bother with parliament or elections (2.11; sd = 0.99). However, the graph also reveals that there is significant variation around these mean preferences—that is, they are not universally shared. Humanity is most unanimous when it comes to viewing democracy as the best form of government (around 90 percent of the world’s population), with a spread that is three-quarters of what we see around the other survey items.

Moreover, results show that the distributions of support for strong leaders or male leaders are more dispersed than support for democracy; they also are notably different from one another. Thus, while a preponderance of humanity thinks that having undemocratic leaders is a bad thing, there is a visible cluster of people who disagree. In fact, around 35 percent of the world’s population consider stronger leaders to be fairly or very desirable. Similarly, opinions are more varied about the suitability of male or female leaders than opinions about the desirability of democracy. There are almost as many humans who agree with the statement that men make better leaders than women (45 percent) than those who disagree with this statement (55 percent).

### Trends in Human Beliefs

**Figure 4.**
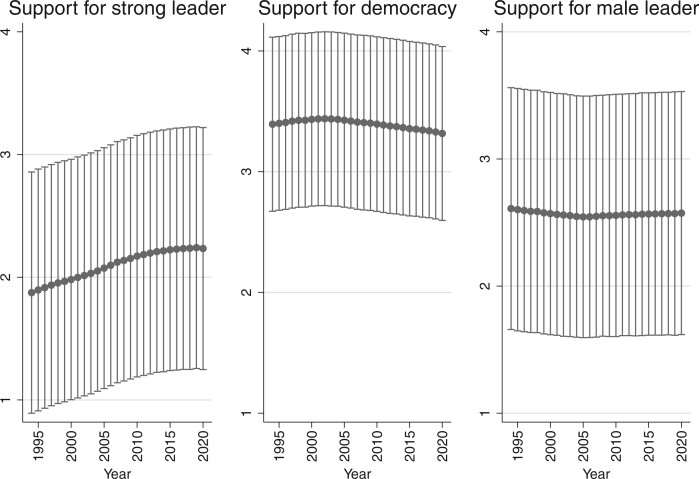
**Trends in preferences.** Dots are means; lines are +1/−1 standard deviations around the means.

We also examined whether these preferences have remained stable over the past two and a half decades. Figure  4 shows the results by year, with the dots representing average responses and the bars representing +1 and −1 standard deviations around the means.^14^ Several things stand out. First, the preference for democracy is stable over time; democracy is humanity’s default governance option. Though currently lower than at its peak around 2000, there is no sign that humanity is moving away from it. The two other survey questions show some trends since the early 1990s. While there is a small decrease in the average human’s willingness to say that men make better leaders (at least until 2005, then the indicator becomes stable), there also is a noticeable increase in support for the idea that having a political system led by a strong, undemocratic leader is a good thing. This increase is linear over the entire period 1994–2020. Clearly, even though humanity says that democracy is the best system, a greater proportion of humans alive today are comfortable with strongman (or -woman) leadership than was true two and a half decades ago.

### Demographic Patterns: A Common Humanity?

**Figure 5.**
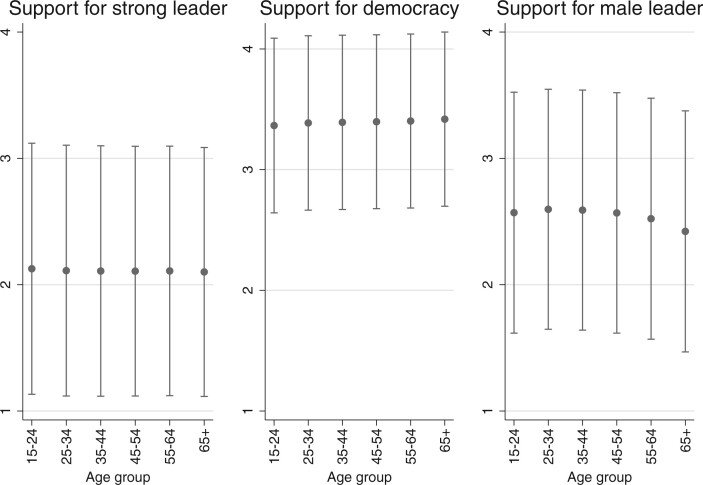
**Preferences by age cohort.** Dots are means; lines are +1/−1 standard deviations around the means.

**Figure 6.**
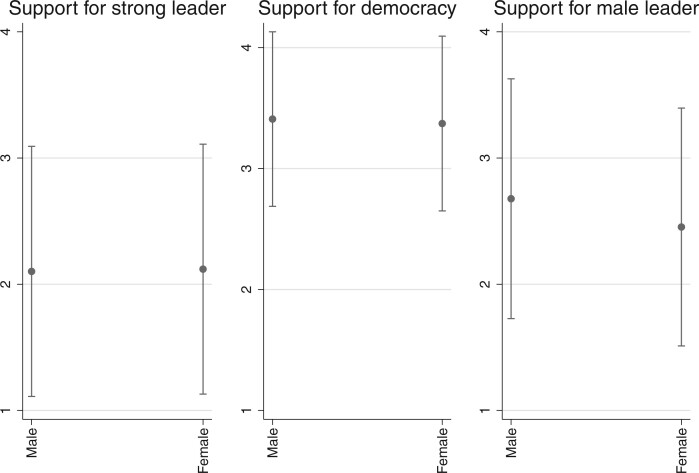
**Preferences by gender.** Dots are means; lines are +1/−1 standard deviations around the means.

**Figure 7.**
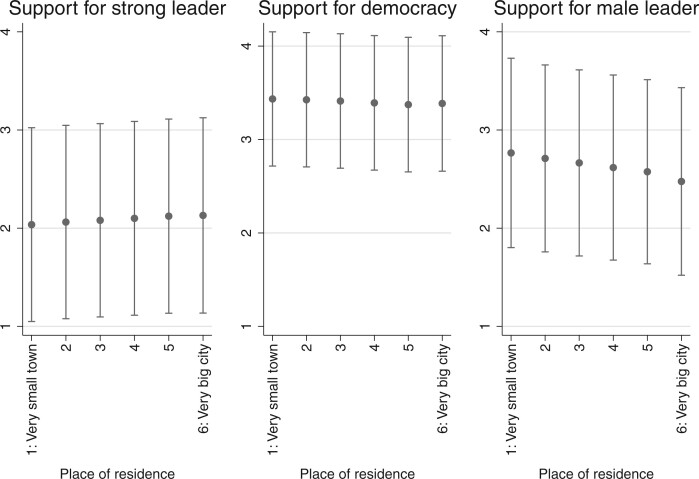
**Preferences by place of residence.** Dots are means; lines are +1/−1 standard deviations around the means.

To see if there are meaningful differences in people’s preferences depending on who they are or where they live, we also calculated survey responses across several demographic groups and world regions. Figures  5–7 show opinions by age group, gender, and place of residence—among the most obvious and widely studied individual differences between human beings on the planet aside from ethnicity.^15^ Perhaps the most interesting result is what we do *not* observe in these graphs: there are only small differences in preferences for political institutions and leaders across the generations, between men and women, or between people living in small towns and big cities. The only apparent, though small, differences are in the views that rural and urban residents, as well as men and women, have about the gender of political leaders, with urban residents and women slightly more likely to disagree with the statement that men make better political leaders. Thus, when humanity as a whole, rather than individual countries, is the relevant sampling frame for analysis, there are few differences between people with different demographic characteristics.^16^

### Humanity’s Preferences: Location, Location, Location?

**Figure 8.**
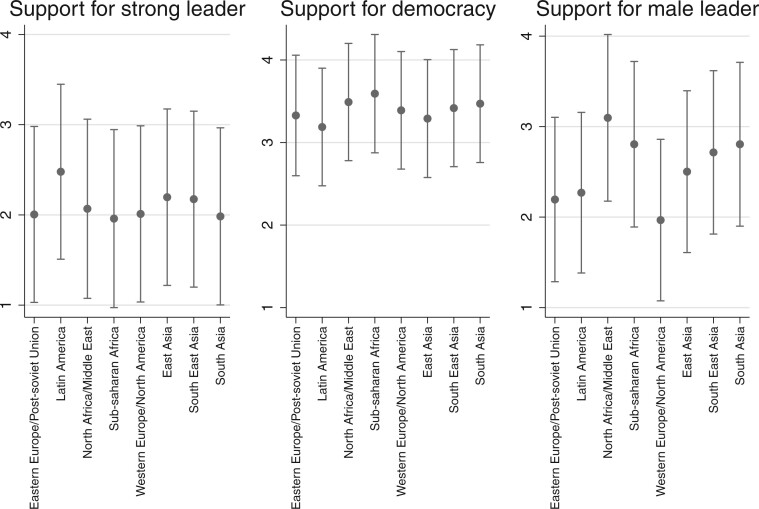
**Preferences by world region.** Dots are means; lines are +1/−1 standard deviations around the means.

While globalizing tendencies have led some to argue that a world polity has been emerging, others have pointed to the strengthening of regional cooperation across the major world regions (Beckfield 2010). Thus, regional organizations like the EU, ASEAN, and the African Union reflect an expansion in structure and policymaking capacity to accommodate greater cross-national integration and cooperation, facilitated by common interests, but also shared political, economic, and social legacies. To see if these legacies leave a lasting impact and shape people’s preferences for democracy and leaders, we also calculated results by world region; these are shown in figure 8.^17^

The results reveal meaningful variation in political preferences across world regions, but primarily with regard to the question of the preferred kind of political leadership. The middle panel in figure 8 shows that support for democracy is high across the board—we see some differences between Latin America at the low end and sub-Saharan Africa at the high end, but these are small, as is the variation around the means. In contrast, we see notable differences across and variations within world regions when it comes to the other two questions. Most notably, there is greater support for the idea that men make better political leaders in North Africa and the Middle East than anywhere else in the world, and this difference is most pronounced compared to Western Europe, where the average respondent is more likely to disagree. Interestingly, variation is also greatest across North Africa and the Middle East compared to the other regions.

Interestingly, Latin American respondents are also more likely to disagree with the notion that men make better political leaders, perhaps bolstered by the demonstration effect of having a number of women serve as presidents of various and big Latin American countries over the years (Argentina, Brazil, Chile, etc.). Finally, support for having a strong leader is highest in Latin America, too, alongside South Asia. Again, we speculate that this may be a function of these regions’ political legacies, though there is clearly a chicken-and-egg issue with regard to whether these preferences are culturally embedded and thus exogenous or endogenous to political developments and path dependent.

**Figure 9.**
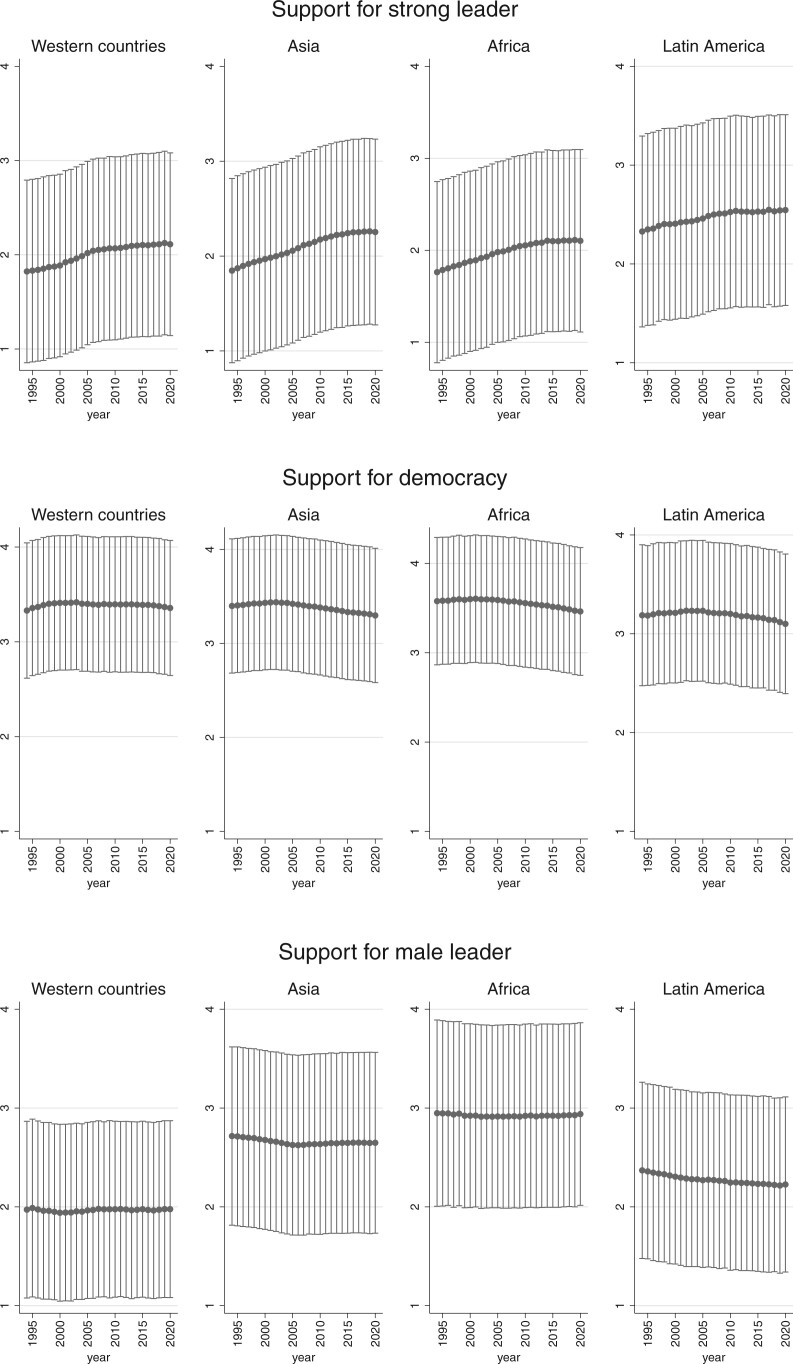
**Trends in preferences by world region.** Dots are means; lines are +1/−1 standard deviations around the means.

Finally, figure 9 shows the trends by region. To facilitate the readability of results, we grouped Asian regions together (East Asia, South Asia, and Southeast Asia), as well as “African” regions (sub-Saharan Africa and North Africa). We report the values for these continents alongside Western countries (Western Europe, North America, Australia, and New Zealand) and Latin America. Although support for democracy seems once again quite stable in all four parts of the world (with a very small decrease in Africa and Latin America), we also find some differences between regions. First, the upward trend in support for strong leaders does not have the same pace everywhere. Whereas it comes to a halt after 2007 in Western countries and in Latin America, it continues to grow somewhat after that in Asia and Africa. Second, it seems that Asians and Africans are much more likely to disagree when it comes to preference for male over female leaders, as captured by the standard deviation, than Westerners and Latin Americans. This is another piece of evidence of the importance of historical development and legacies in political attitudes regarding leaders.

**Figure 10.**
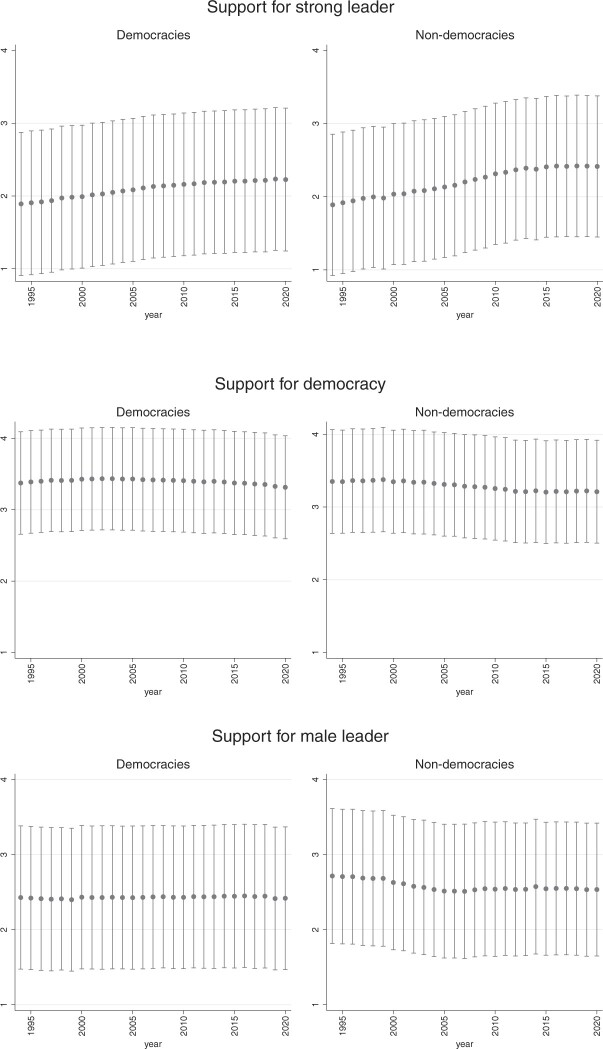
**Trends in preferences by regime type.** Dots are means; lines are +1/−1 standard deviations around the means.

Finally, following the line of work of Claassen (2020b) and Inglehart and Welzel (2005), we examine preferences across regime types. We expect that the political conditions under which people live affect their stated preferences. In particular, we examine whether the preferences of citizens living in a nondemocratic and democratic countries differ significantly.^18^  Figure  10 shows that humans who are experiencing democracy in their daily life are as supportive as those who live in less democratic countries. Yet, we also see that the upward trend in support for strong leaders between 1995 and 2010 is notably steeper in nondemocracies than in democracies. Echoing the thermostatic model of democratic preferences developed by Claassen, this finding suggests that real-life political conditions under which people live do affect their preferences and constitute a benchmark against which they evaluate the performance and desirability of regimes. Interestingly, the preferences of citizens living in less democratic countries are essentially similar to those living in democratic ones when it comes to believing that men and women make equally good leaders. Finally, it is worth noting that the degree of disagreement is larger in democracies than in nondemocracies with regard to all three outcome variables. We speculate that this may be due to a greater willingness to express views that deviate from majority opinion in more democratic states because of laws and norms that protect and value freedom of speech and conscience in these countries.

## Conclusion

What do humans think about democracy and political leaders? Moreover, does humanity agree or is it divided when it comes to such fundamental issues of governance? While these questions are both simple and important, they currently do not have solid empirical answers. In this paper, we therefore attempt to address them by developing a new empirical approach that can be used with existing data from around the world to fill in the *terra incognita* previously not charted by cross-national surveys and generate the average human’s response to various questions about politics and democracy.

We find that humans almost universally prefer democracy as a system of government, but there are also growing signs that they are becoming more accepting of government by strong leaders who are unencumbered by democratic institutions and processes. These signs are especially notable and growing in regions of the world where democracy is in danger of backsliding or has not fully taken hold. Citizens in many third-wave democracies, for example, still face challenges from authoritarian elements. Thus, depending on their success in fending off these threats, they would hold divergent perceptions or even skepticism of democratic and liberal values.

Interestingly, attitudes about democracy, strong leaders, or the gender of political leaders vary less across people’s demographic characteristics than the region of the world where respondents live. The fact that there are relatively small differences based on individual characteristics of course does not preclude the possibility that bigger differences exist across other individual characteristics or are more or less consequential within certain countries or regions. But it does suggest that, once we strip away macro-level or cultural differences, people are similar in what they believe. There seems to be a common set of beliefs about governance that divides humanity primarily by structural conditions or world region rather than differences across the old and young or men and women.

A positive piece of news from our study is the confirmation of existing research that support for democracy is almost universal. Moreover, despite a lively scholarly and public debate about a global democratic recession, the preference for democracy as a system of government is holding steady globally and has for well over two decades. While there are signs of democratic backsliding in specific countries, this has taken place within a broader context of stable global preferences for democratic government. At the same time, our results suggest that, as international and transnational politics becomes regionalized, there are distinct regional clusters of democratic preferences.

For those who advocate greater equality in politics, our findings contain both bad and good news—bad, because the data show that humans tend to lean slightly toward the belief that men make better leaders, and this is consistent with the pattern of actual officeholders we see in the world. While there is a suggestion that the human preference regarding the gender of political leaders has been changing over the past two decades, with a steady downward tick in people’s expressed views that men make better political leaders, the only world region where a majority of people disagree is Western countries, while elsewhere, people’s revealed preference for political leaders is still heavily skewed toward men.

Finally, we hope our study serves as a blueprint for future studies. On an empirical and methodological level, our approach can easily be replicated to examine other questions or specific regions of the world (including within-nation differences) where survey data may be available but are currently incomplete. It will allow researchers to fill in a number of blank spots, perhaps most obviously when specific world regions or continents are the subject of investigation. We hasten to add that this does not mean we no longer need to collect survey data; after all, our approach requires good cross-national coverage of countries and regions. Moreover, none of our computations are designed to predict future survey responses and of course cannot account for the impact of exogenous events (e.g., war, natural disasters, or regime change).

As with any survey project aimed at gauging what the global public thinks, there is potentially a trade-off between asking specific questions about politics and ascertaining views that are meaningful on a global scale. We suspect that this trade-off may be most easily overcome with regard to gauging people’s opinions about essential elements of the human experience, such as physical and subjective well-being, security, and the like, or the role of social relations and religion, to name a couple of examples. With regard to political views in particular, we imagine that especially issues that are universal, transcend boundaries, or require cross-border state-level interaction and cooperation would be potentially of greatest interest—for example, questions around the environment, climate change, and migration but also issues such as gender equality, food security, or global (distributive) fairness, for instance.

Second, and on substantive grounds, while our results make no assumptions about the existence of a “global public,” we can think of them as establishing a *baseline of beliefs* that members of our species hold. By providing information about how all people feel about public matters, we hope to contribute to the measurement of the human condition. Of course, one important normative question is whether thinking about people as equals—where each person has equal weight—is the right way to think about the global population in the context of an emerging global demos, or whether we should think of this global public as constituted of many national and unequal publics.

## Supplementary Material

nfab056_Supplementary_Data

## Data Availability

REPLICATION DATA AND DOCUMENTATION are available at https://doi.org/10.7910/DVN/KSEG9Y.

## Footnotes

1 We bracket the question of the meaning of democracy in nondemocratic countries (see, e.g., Kirsch and Welzel 2019). Unfortunately, detailed questions about people’s interpretations of democracy were only recently introduced into the WVS.

2 Notably, these included, among others, the Eurobarometer Surveys, European Social Surveys, Latin America Public Opinion Project, WVS, or International Social Survey Project (Kittilson 2007; Norris 2009).

3 Note that the estimation of a latent estimate based on several survey questions can be an advantage, especially when the question or response scale is poorly worded. In that situation, the combination of several questions can help reduce the noise or bias brought by an imprecise survey question. For examples of research in which answers to a single question are imputed to fill the gaps between survey waves or to estimate values prior to the first survey wave, see Dahlum and Knutsen (2017) and Ruck, Bently, and Lawson (2018), respectively.

4 Our analyses are meaningful to the extent that the survey instruments are reliable and valid—perhaps most importantly, that there is reasonable equivalence in the meaning of terms across countries, cultures, and languages (Stegmueller 2011). One way of testing the validity of the survey questions is to see whether answers are correlated with phenomena they should be related to (predictive validity). Indeed, this is what Claassen’s (2020a) study of the link between support for democracy and democratization and democratic stability does. He finds that support for democracy predicts democratic stability (though not the emergence of democracy). Thus, even though people’s and societies’ definitions and understandings of the concept of democracy differ (Davidov et al. 2014), survey responses have the power to predict real-world phenomena. We bracket other potential validity problems, including potential over- or underreporting of true preferences, for example, because of a fear of repression, social desirability bias, or misunderstanding the survey question (Ananda and Bol 2021; Kruse, Ravlik, and Welzel 2019). These are not issues we can address with the data available to us. Note, however, that some surveys were excluded due to documented translation problems (Kurzman 2014).

5
http://www.worldometers.info/world-population/world-population-by-year/. We proceed by WVS wave, and so use the average world’s population over the period (between 4.5 and 7.3 billion). Since the WVS waves cover periods of four to six years, we use the average number of UN countries during the period (between 167 and 193).

6 Although intuitively appealing, the strategy of having each line representing a single human being would not be efficient for reasons related to computing power. The number 10,000 is a reasonable threshold because there is only one country with a population that falls below (the Vatican). However, it would be easy for researchers with access to a more powerful computer to adapt our code to increase the granularity.

7 Studies that use MRP usually skip this first step and create a dataset with synthetic citizens that reflects the demographics of the geographic unit of interest as they appear in official statistics. Although simpler, we decided against this strategy for two reasons. First, there are no official statistics for all of the sociodemographic variables we wanted to include. Second, we wanted to account for correlations between these sociodemographic variables. This is especially important when working with data from developing countries where there are often strong correlations between sociodemographic variables (e.g., between gender and education level). We therefore use individual-level data from the WVS to construct our humanity dataset. In the Supplementary Material, we show that our simulation results are very similar to available official statistics for the covariates.

8 In the literature on machine learning, this is often referred to as a “bias-variance trade-off” that is especially important for our analysis, as our goal is to undertake out-of-sample predictions for countries not covered in the WVS. Note that using an atheoretical method to select the set of covariates that maximizes the parameters of this trade-off, like Lasso regression, would not change the results (Broniecki, Leeman, and Wuest 2020). As the results below show, the regressions make excellent in-sample and out-of-sample predictions.

9 To see how this works in practice, an example may help fix ideas: Imagine a country with a population of 50,000,000, where our regression predicts that the income distribution has a mean of 5 and a standard deviation of 2. We then generate 5,000,000 random synthetic observations (1 line for 10,000 human beings) where the mean of the income variable is 5 with a standard deviation of 2. Further, imagine that our regression predicts that education has a mean of 4 and a standard deviation of 3, and correlates with income at 0.8. We then randomly generate the variable education so that the mean is 4, the standard deviation is 3, and the correlation between the two generated variables income and education is 0.8. We do this for all countries of the world, for each year between 1994 and 2020, and for all five sociodemographic characteristics.

10 We included the following interaction terms: education (individual) x corruption level (country), education (individual) x democracy (country), income (individual) x corruption (country), income (individual) x democracy (country), gender (individual) x percent of Catholics (country), gender (individual) x percent of Muslims (country), gender (individual) x percent of Protestants (country), and age (individual) x year (country).

11 Including country fixed effects would defeat the purpose of the analysis. The objective is indeed to impute values of the outcome variables for countries not included in the WVS, and then not in the dataset. For the same reason, we cannot include lagged covariates or lagged outcome variables.

12 Concretely, we compute 10 iterations of multiple imputations. Due to limitation in computing power, we cannot use all 10 of them. Instead, we randomly select one iteration for each human being in the humanity dataset and consider the corresponding prediction as the imputed value.

13 The question asks whether respondents agree with the statement that “men make better political leaders than women.” This means that the responses of “agree” or “strongly agree” indicate the belief that men make better leaders. However, the response categories “strongly disagree” or “disagree” do not clearly indicate whether respondents believe women to be better political leaders. Instead, disagreeing with the statement that men make better leaders may simply indicate that respondents see men and women as equally qualified to be leaders.

14 Given our extremely large *N*, reports of standard errors and statistical significance would not be informative. Moreover, given that the estimates are for all of humanity rather than a sample of it, the concept of statistical significance has little relevance.

15 We do not present the results broken down by education, income, and urbanization because the three variables are not perfectly comparable across countries, and hence are less interesting from a global perspective. For example, living in a city of more than 500,000 people does not have the same meaning in China as it does in Belgium. Similarly, education varies globally, including in terms of access and structure of the education system. For an illustration of challenges in creating a standardized education measurement, see Mattes and Mughogho (2009).

16 This lack of differences across demographic groups dovetails results from our multilevel regressions predicting attitudes using the raw WVS data (those we use to construct our estimates). These results, too, show that demographic covariates are only weakly associated with responses to these questions. Specifically, while the coefficients are often statistically significant because the *N* is very large, their substantive impact is very small (around 0.01, 0.02). Similarly, the multilevel regressions produce the largest coefficient for gender and support for male leaders (∼0.30), consistent with our estimates using the humanity dataset. Thus, we find the largest difference in sociodemographics for gender and support for male leader, regardless of data or estimation method.

17 The variable “region of the world” is taken from the QOG dataset using the classification of Hadenius and Teorell (2007). Note that we excluded the category Pacific and “Caribbean” because it contains very few observations compared to the other regions and is therefore very sensitive to outliers.

18 In order to separate democracies from nondemocracies, we rely on the indicator “Liberal democracy” from the VDEM project and included in the QOG data. This indicator is the result of a survey asking experts to locate countries in terms of liberal democracy (i.e., procedural democracy as well as freedom and civil rights). We categorize nondemocracies as those located one standard deviation below the mean and democracies those that are one standard deviation above the mean.

## Notes

Christopher J. Anderson is a professor in Politics and Policy in the European Institute at the London School of Economics and Political Science, London, UK.

Damien Bol is an associate professor of political behavior in the Department of Political Economy at King’s College, London, UK.

Aurelia Ananda is a PhD candidate in the Department of Political Economy at King’s College, London, UK. Earlier versions of this paper were presented at the annual meetings of the Midwest Political Science Association, European Political Science Association, American Political Science Association, and EPOP (Elections, Public Opinion and Parties), as well as the seminar of the Chair for the Study of Democracy at Leuphana University. The authors are grateful to conference and seminar participants, Kathrin Ackerman, André Blais, Matthew Graham, Andrew Philips, and Christian Welzel, as well as the editors and reviewers for their helpful comments on earlier versions of this paper.

## Appendix A. Wording of WVS Questions

The survey data and documentation are available at worldvaluessurvey.org. The question wording of the specific survey questions used in the analysis is as follows.

** Support for a democratic political system:** “I’m going to describe various types of political systems and ask what you think about each as a way of governing this country. For each one, would you say it is: (1) very bad, (2) fairly bad, (3) fairly good, or (4) very good way of governing this country? … Having a democratic political system”

** Support for strong leader:** “I’m going to describe various types of political systems and ask what you think about each as a way of governing this country. For each one, would you say it is (1) very bad, (2) fairly bad, (3) fairly good, or (4) very good way of governing this country? Having a strong leader who does not have to bother with parliament and elections”

** Support for male leader:** “For each of the following statements, can you tell me how strongly you agree or disagree with each. Do you (1) strongly disagree, (2) disagree, (3) agree, or (4) strongly agree? On the whole, men make better political leaders than women do”

** Gender:** [respondent’s sex coded by observation: *male* or *female*]

** Education:** At what age did you (or will you) complete your full-time education, either at school or at an institution of higher education? Please exclude apprenticeships. [*NOTE: If respondent indicates to be a student, code highest level s/he expects to complete.]*

_______ (*write in age in two digits*)

(Code number): (Lowest group) 1 2 3 4 5 6 7 8 9 10 (Highest group)

** Income:** On this card is an income scale on which 1 indicates the lowest income group and 10 the highest income group in your country. We would like to know in what group your household is. Please, specify the appropriate number, counting all wages, salaries, pensions and other incomes that come in.

(Code number): (Lowest group) 1 2 3 4 5 6 7 8 9 10 (Highest group)

** Age:** This means you are ______ years old (*write in age in two digits*)

(Code number): (Lowest group) 1 2 3 4 5 6 (Highest group)

**Table:**

**Urbanization:** (*Code size of town*):	1) Under 2,000
	2) 2,000–5,000
	3) 5–10,000
	4) 10–20,000
	5) 20–50,000
	6) 50–100,000
	7) 100–500,000
	8) 500,000 and more

## Appendix B. Descriptive Statistics for Quality of Government Dataset

**Table B1.** QOG variables used in the analysis (1994–2020)

Variable	*N*	Mean	Std. dev.	Min	Max
Population					
Total population	8,568	3.11E + 07	1.18E + 08	6,337	1.40E + 09
% Population female	8,165	50.00	2.62	23.29	55.63
% Population <14	8,165	33.94	10.73	12.21	51.64
% Population >65	8,165	6.55	4.75	0.69	28.00
Country development profile					
Democracy index (VDEM)	8,979	0.34	0.28	0.00	0.89
Corruption index (VDEM)	8,989	0.49	0.30	0.01	0.97
GDP per capita	4,587	15,107	18,234	325	141,635
% Pop. with telephone	8,059	13.98	17.62	0.00	135.60
Latitude (absolute)	10,362	0.26	0.18	0.00	0.72
% Urban population	8,569	50.69	24.85	2.19	100.00
% Catholic population	10,164	34.00	36.67	0.00	99.10
% Muslim population	10,164	22.59	35.50	0.00	99.90
% Protestant population	10,164	13.05	21.08	0.00	97.80
