# Supplementary material for: Humanity’s Attitudes about Democracy and Political Leaders: Patterns and Trends
Source: Public Opin Q. 2021 Nov 26;85(4):957–86. doi: 10.1093/poq/nfab056 (PMC8754486; doi:10.1093/poq/nfab056)
Supplement: nfab056_Supplementary_Data [file nfab056_supplementary_data.pdf]

Supplementary Material

Humanity's Attitudes About Democracy and Political Leaders:

Patterns and Trends

Christopher J. Anderson
European Institute
London School of Economics and Political Science
Houghton Street, London WC2A 2AE, United Kingdom
Email: c.anderson7@lse.ac.uk
(Corresponding author)

Damien Bol
Department of Political Economy
King's College London
Strand, London WC2R 2LS, United Kingdom
Email: damien.bol@kcl.ac.uk

Aurelia Ananda
PhD Candidate
Department of Political Economy
King's College London
Strand, London WC2R 2LS, United Kingdom
Email: aurelia.nugroho@kcl.ac.uk

- A. Step One: Creating a Humanity Dataset
- B. Step Two: Simulating What Humanity Thinks

The Supplementary Material contains the validity tests of the first step (Part A) and the second step of our analysis (Part B).

A. Step One: Creating a Humanity Dataset

To assess the validity of the humanity dataset, we first conduct a series of out-of-sample tests to verify that the OLS regression presented in the main text (see Equation 1) can accurately predict the parameters of the joint socio-demographic distributions for countries and years *not* included in the regression. To do so, we randomly select a subset of observations among those available the WVS, which we call the "test sample". We estimate the regression without these observations and check whether the predictions are close to the actual values of the test sample. We perform this exercise with two types of test samples: one that randomly selects and removes 45 country-years, and one that randomly selects and removes all the years from 15 countries. These numbers imply that we remove about 30% of the observations of the dataset. This proportion corresponds to the proportion of countries for which we want to impute a value in the main analysis of the paper, i.e., the population of countries covered in the WVS compared to the population of countries of the world (see Table 1 in the main text). Furthermore, to minimize the probability that the results are due to chance, we run a Monte Carlo simulation that reproduces the same two out-of-sample exercises 20 times on 20 different and randomly-generated test samples.

Table SM1 presents the results. They show that the average difference between the predictions and the actual values in the test samples for each of the parameters of the joint distributions is very small. For means and standard deviations, average predictions often recover actual values with a precision of 0.10, and not greater than 0.50. This is very small, given that most variables vary between 1 and 6 or 1 and 10 (see Appendix A in the main text). For correlations, which vary between -1 and 1, the difference between predictions and actual

values is rarely larger than 0.05. This means that the regressions are able to recollect the value of parameters of countries and country-years not included in the regressions.

Table SM1. Results of the out-of-sample tests (humanity dataset)

Variables	Test Sample 45 random country-years	Test Sample 15 random countries
Female (0-1) (mean)	<0.01 (<0.01)	<0.01 (0.01)
Female (0-1) (standard deviation)	<0.01 (<0.01)	<0.01 (<0.01)
Education (1-10) (mean)	0.18 (0.26)	-0.07 (0.06)
Education (1-10) (standard deviation)	<0.01(0.14)	0.09 (0.02)
Age (1-6) (mean)	0.02 (0.08)	-0.04 (0.04)
Age (1-6) (standard deviation)	<0.01 (0.01)	-0.04 (0.02)
Urbanization (1-6) (mean)	0.13 (0.29)	0.06 (0.18)
Urbanization (1-6) (standard deviation)	0.06 (0.17)	-0.14 (0.13)
Income (1-10) (mean)	0.06 (0.08)	0.14 (0.01)
Income (1-10) (standard deviation)	<0.01 (0.10)	0.03 (0.01)
Female x Education (correlation)	0.02 (0.02)	0.01 (0.03)
Female x Age (correlation)	0.01 (0.01)	<0.01 (0.01)
Female x Urbanization (correlation)	<0.01 (<0.01)	0.01 (<0.01)
Female x Income (correlation)	0.01 (0.01)	0.01 (0.01)
Education x Age (correlation)	0.02 (0.02)	0.01 (0.01)
Education x Urbanization (correlation)	0.02 (0.02)	0.06 (0.06)
Education x Income (correlation)	0.06 (0.06)	<0.01 (<0.01)
Age x Urbanization (correlation)	0.01 (0.01)	<0.01 (<0.01)
Age x Income (correlation)	0.02 (0.02)	0.01 (0.01)
Urbanization x Income (correlation)	<0.01 (0.02)	0.02 (0.01)

Note: Entries are average differences between predictions from OLS regressions and actual values in the test sample over 20 simulations. Standard deviations over the same 20 simulation are in parentheses.

To further assess the reliability of the humanity dataset, we examine the correlations between the humanity dataset and official statistics. Due to data limitation, the only socio-demographic characteristic for which we can perform this test is age. We have official statistics about the proportion of people between 16 and 64 years old in each country and years covered in the humanity dataset. We can thus compare official statistics with the humanity dataset, separating country-years included in the WVS (and thus used to estimate the regressions used to create the humanity dataset), country-years not included in the WVS, and country-years of countries never included in any of the waves of the WVS. Figure SM1 shows that the correlations for the different age categories are very high, both for countries and years included in the WVS and those that are not.

Figure SM1. Correlations between humanity dataset and official statistics

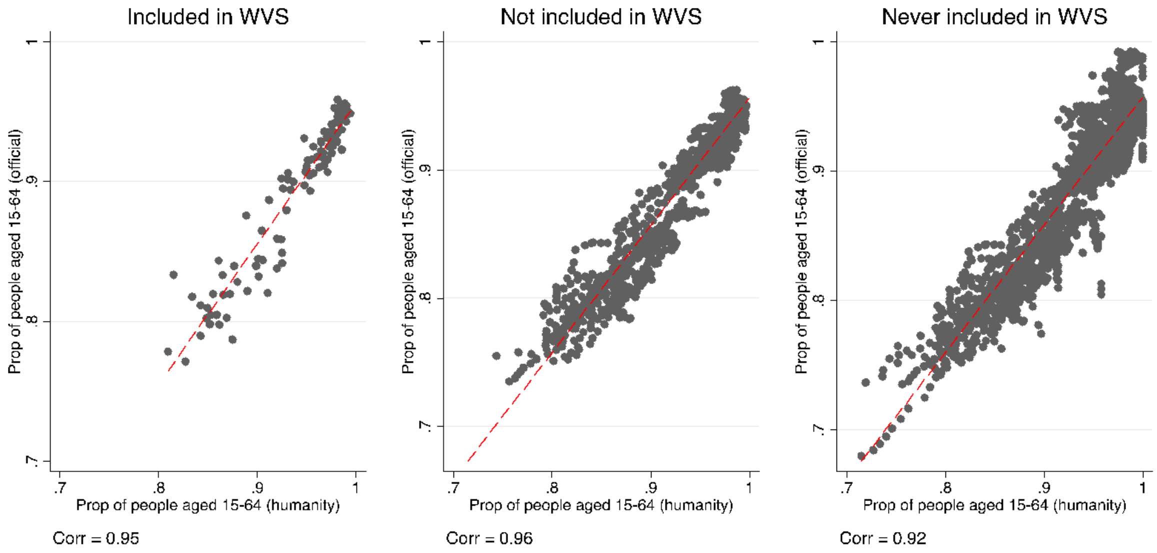

Note: Left panel includes country-years included in the WVS. Middle panel includes country-years not included in the WVS. Right panel includes country-years from countries never included in the WVS.

As a final and perhaps more intuitive example of evidence for the quality of our humanity dataset, we report key correlations between socio-demographic variables from four selected countries: Mozambique, Nigeria, Belgium, and the Netherlands. The first two share low UN Human Development Index scores, whereas the latter two score highly on the index. Also, note that one country in each group is covered by the WVS (Nigeria and Netherlands), whereas the other one is not (Mozambique and Belgium). We can thus compare them to evaluate how good the simulation of the humanity dataset is.

We show correlations that relate to the state of equal opportunities since we have strong priors regarding the differences between low and high human development-countries. The results, shown in Table SM2, show that opportunities are more equal in Belgium and the Netherlands than in Mozambique and Nigeria, with education level more highly (negatively) correlated with female gender in the latter (between -0.09 and -0.07) than in the former (between -0.01 and -0.04). Note however that the correlation between education and income is similar across all four countries (between 0.18 and 0.37). Overall, these analyses provide support for the quality of the humanity dataset, and especially the quality of the synthetic samples relative to those covered by the WVS.

Table SM2. Correlations in humanity dataset for selected countries

	**Mozambique** (Not in WVS)	**Nigeria** (WVS)	**Belgium** (Not in WVS)	**Netherlands** (WVS)
Female and Education	-0.09	-0.07	-0.03	-0.01
Income and Education	0.25	0.18	0.37	0.27

Note: Entries are correlation coefficients.

To conclude, Table SM 3 describes the humanity dataset. It shows the means and standard deviations (sd) of the five socio-demographic characteristics, as well as their correlations. Given that there are around 4.5 billion human beings over the age of 15 in the

countries covered in the analysis (~90% of the world's population) and given that each line in our dataset represents 10,000 individuals and that we cover 27 years, the number of observations is around 12 million. The table reveals some important patterns in the correlations between the socio-demographic characteristics. First and unsurprisingly, the strongest correlations are between education and age (-0.20), and education and income (0.21). Second, urban residence is positively correlated with levels of education and income (0.09 and 0.10 respectively). Finally, female is uncorrelated with any of the other variables except for education (-0.06). The lack of a correlation between the variables "female" and "income" is likely due to the fact that income is measured at the level of households rather than individuals. Overall, the descriptive statistics of humanity confirm conventional wisdom regarding the distribution of socio-demographics in the world.

Table SM3. Summary of socio-demographic variables in the humanity dataset

	Female	Education	Age	Urban	Income
**Female** (0-1)<br>Mean=0.50, sd=0.50	1.00				
**Education** (1-10)<br>Mean=6.48, sd=2.54	-0.06	1.00			
**Age** (1-6)<br>Mean=3.14, sd=1.39	-0.02	-0.20	1.00		
**Urban** (1-6)<br>Mean=4.70, sd=1.42	<0.01	0.09	0.01	1.00	
**Income** (1-10)<br>Mean=4.61, sd=2.00	-0.02	0.21	-0.05	0.10	1.00

Note: Cell entries are correlation coefficients.

B. Step Two: Simulating What Humanity Thinks

To assess the validity of the second step, we first report the results of the multi-level OLS regression presented in the main text (see Equation 2), which predicts the three outcome variables of interest (Table SM4). While most coefficients are not statistically different from zero, recall that the aim of the regressions is not to look for significant coefficients but to see if we can use information about countries and respondents to predict opinions both in the countries and years for which we have survey data and those for which we do not. Thus, it would not be appropriate to remove the covariates that do not achieve statistical significance from the regression.

Table SM4. Multilevel OLS regressions predicting responses to survey questions

	**Strong Leader**	**Democracy**	**Male Leaders**
Total pop. (log)	0.02	-0.04	0.02
	(0.33)	(0.00)	(0.23)
% pop. female	-0.01	-0.04	0.04
	(0.85)	(0.12)	(0.19)
% pop. <14	-0.00	0.01	0.01
	(0.64)	(0.00)	(0.03)
% pop. >65	-0.03	0.04	-0.02
	(0.25)	(0.00)	(0.07)
Democracy index	0.02	-0.35	-0.16
	(0.96)	(0.05)	(0.35)
Corruption index	-0.31	-0.02	-0.12
	(0.34)	(0.90)	(0.51)
GDP per capita	0.15	-0.06	0.05
	(0.17)	(0.23)	(0.53)
% pop. with telephone	-0.01	0.00	-0.00
	(0.09)	(0.12)	(0.08)
Latitude (absolute)	-0.27	-0.41	0.17
	(0.29)	(0.00)	(0.33)
% urban pop.	0.01	-0.00	0.00
	(0.05)	(0.20)	(0.61)
% Catholic pop.	0.00	-0.00	-0.00
	(0.62)	(0.03)	(0.00)
% Muslim pop.	-0.00	0.00	0.00
	(0.03)	(0.03)	(0.00)
% Protestant pop.	-0.00	0.00	-0.01
	(0.24)	(0.59)	(0.00)
Year	2.10	1.76	-0.57
	(0.56)	(0.29)	(0.83)

$Year^2$	-0.00	-0.00	0.00
	(0.56)	(0.29)	(0.83)
Female	0.02	-0.04	-0.23
	(0.48)	(0.01)	(0.00)
Education	-0.03	-0.00	-0.04
	(0.18)	(0.66)	(0.01)
Age	-0.14	1.11	4.47
	(0.95)	(0.48)	(0.01)
Urban	-0.00	0.01	-0.01
	(0.57)	(0.21)	(0.09)
Income	-0.00	-0.02	-0.03
	(0.97)	(0.33)	(0.10)
Education * Corruption index	0.03	0.00	0.02
	(0.10)	(0.68)	(0.10)
Education * Democracy index	-0.02	0.03	0.01
	(0.47)	(0.00)	(0.40)
Income * Corruption index	0.02	0.01	0.02
	(0.57)	(0.49)	(0.30)
Income * Democracy index	-0.02	0.04	0.01
	(0.59)	(0.06)	(0.52)
Female * % Catholic pop.	-0.00	0.00	-0.00
	(0.15)	(0.71)	(0.20)
Female * % Muslim pop.	-0.00	0.00	-0.00
	(0.71)	(0.24)	(0.01)
Female * % Protestant pop.	-0.00	-0.00	0.00
	(0.58)	(0.63)	(0.44)
Age * Year	0.00	-0.00	-0.00
	(0.96)	(0.49)	(0.01)
Observations	91,241	103,444	107,727
R-squared	0.08	0.06	0.21

Note: Entries are coefficient estimates from OLS regressions. P-values (two-tailed, standard errors clustered by country/year) are in parentheses.

To further test the reliability of the second step, we conduct another series of out-of-sample tests. As in the previous one, we randomly select and remove a subset of observations, which we call “test sample”, before estimating the regression. Also as in the previous one, we rely on two test samples: one in which we randomly select and remove 45 country-years, and one in which we randomly selected remove all the years from 15 countries. These represents about 30% of the entire dataset and more than 50,000 individual respondents. Figure SM2 reports the difference between prediction and actual values in the test sample for each of the 20 Monte Carlo simulations. It shows that the regression is often capable of retrieving the right

value with a precision of 0.1. The difference is never greater than 0.4. This is very small given that the outcome variables have a response scale varying between 1 and 4.

Figure SM2. Out-of-sample prediction tests, all 20 simulations

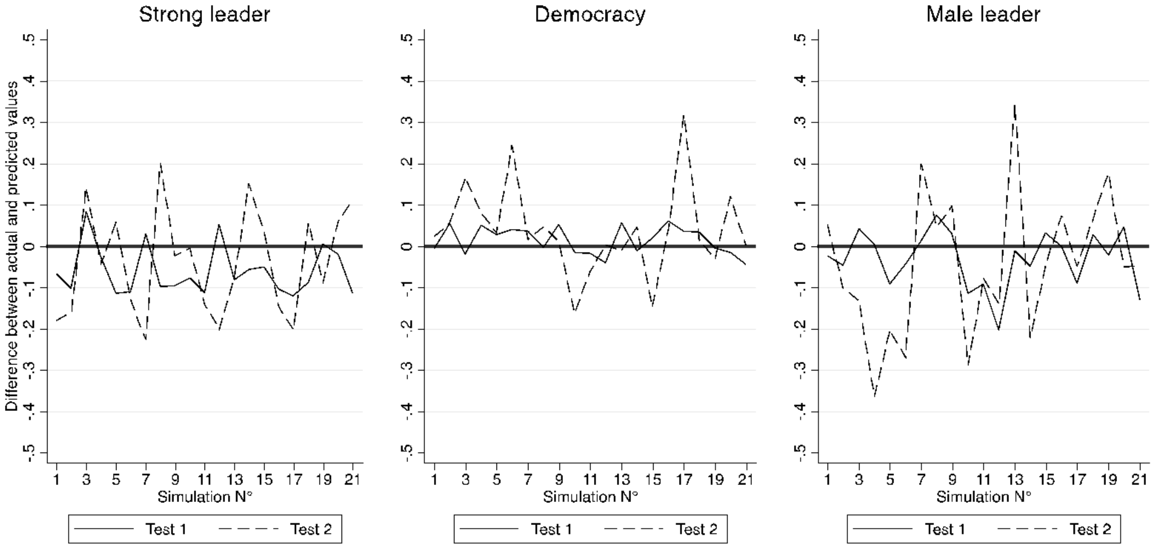

Note: Test sample in Test 1 consists of 45 randomly-removed country-years. Test sample in Test 2 consists of 15 randomly-removed countries (all years).

Third, we check whether countries that have never been included in the WVS are systematically different from those that have been. This test is important because it evaluates whether the countries that are missing in the WVS dataset are “missing at random”. The out-of-sample tests presented randomly exclude some countries to form the test sample. Because this exclusion is random, the countries of the test sample are necessarily similar to others. We already know that the regression performs well in this situation, but it does not mean that it would perform well if the countries in the test sample were systematically different from others. Predictions on extreme counterfactuals (i.e., observations for which the value of covariates is very different from those in the sample) are always hazardous and not especially accurate (King and Zeng 2006).

To check this assumption, we perform two tests. In Table SM5, we report bivariate t-tests estimating whether countries that have never been included in the WVS and those that have been has a different mean on the country-level variables included in our analysis (see Appendix B in the main text). Table SM6 reports the results of a multivariate OLS regression predicting the inclusion of a country in the WVS (vs. never included) by the same country-level variables. Both tests show that, although the two sets of countries are slightly different, in particular countries included in the WVS are more populous and urban, as well as generally more developed (less children, greater access to telephone), such differences are small – for example, the difference in means is never greater than half of the mean itself.

Table SM5. Balance test (univariate)

	**Strong Leader**			**Democracy**			**Male Leaders**		
	Not WVS	WVS	Diff	Not WVS	WVS	Diff	Not WVS	WVS	Diff
Total pop. (log)	14.12	16.51	-2.39	14.05	16.56	-2.50	14.03	16.52	-2.49
% pop. female	49.60	50.58	-0.98	49.62	50.55	-0.93	49.87	50.32	-0.45
% pop. <14	38.25	29.89	8.36	38.29	29.95	8.32	38.37	30.05	8.32
% pop. >65	4.82	8.18	-3.35	4.83	8.14	-3.30	4.89	8.01	-3.12
Democracy index	0.31	0.44	-0.12	0.31	0.44	-0.13	0.32	0.43	-0.11
Corruption index	0.54	0.52	0.03	0.55	0.52	0.03	0.55	0.52	0.03
GDP per capita (log)	8.31	8.69	-0.39	8.32	8.68	-0.37	8.29	8.70	-0.41
% pop. with telephone	13.83	20.83	-7.00	13.95	20.63	-6.68	13.90	20.55	-6.65
Latitude (absolute)	0.22	0.31	-0.09	0.22	0.31	-0.09	0.21	0.31	-0.09
% urban pop.	46.46	58.57	12.11	46.66	58.24	11.58	45.79	58.83	13.04
% Catholic pop.	32.24	36.00	-3.76	32.62	35.53	-2.91	33.43	34.60	-1.17
% Muslim pop.	22.84	22.30	0.54	22.98	22.15	0.83	21.14	24.11	-2.97
% Protestant pop.	14.95	10.88	4.06	15.12	10.75	4.37	15.49	10.48	5.01

Note: Entries are means and differences in means.

Table SM6. Balance test (multivariate)

	Strong Leader	Democracy	Male Leaders
Total pop. (log)	0.12	0.14	0.15
	(0.00)	(0.00)	(0.00)
% pop. female	0.05	0.04	0.04
	(0.07)	(0.14)	(0.14)
% pop. <14	-0.03	-0.03	-0.02
	(0.01)	(0.01)	(0.03)
% pop. >65	-0.04	-0.04	-0.03
	(0.17)	(0.18)	(0.27)
Democracy index	0.02	0.13	0.05
	(0.94)	(0.62)	(0.85)
Corruption index	0.21	0.18	0.10
	(0.38)	(0.46)	(0.66)
GDP per capita	-0.06	-0.06	-0.02
	(0.47)	(0.45)	(0.82)
% pop. with telephone	-0.00	-0.00	-0.00
	(0.99)	(0.83)	(0.61)
Latitude (absolute)	0.17	0.16	0.16
	(0.61)	(0.64)	(0.63)
% urban pop.	0.01	0.01	0.01
	(0.05)	(0.06)	(0.05)
% Catholic pop.	0.00	0.00	0.00
	(0.49)	(0.66)	(0.71)
% Muslim pop.	0.00	0.00	0.00
	(0.11)	(0.16)	(0.15)
% Protestant pop.	0.00	0.00	0.00
	(0.72)	(0.82)	(0.81)

Note: Entries are coefficient estimates from OLS regressions predicting the inclusion of the country in the WVS (all wave considered) or not. P-values (two-tailed) are in parentheses.

Fourth, we check whether very large countries are accurately predicted by the regression. Given that these countries are very influential on the results because of their size, an inaccurate prediction might bias the global estimates. In Figure SM3, we therefore plot the average value of the outcome variables by country-year in the humanity dataset and in the WVS. In other words, it captures the "fit" of the regression or if the predictions (in the humanity dataset) fit the original values (in the WVS). We systematically identify the five largest countries in the world as of 2020: China, India, United States, Indonesia and Pakistan. All together they represent half of the world's population. We find that these five countries are no

different from others in term of "fit": their predictions in the humanity dataset are as close form the WVS prediction than it is for other countries.

Figure SM3. Prediction in the humanity dataset and actual values in the WVS.

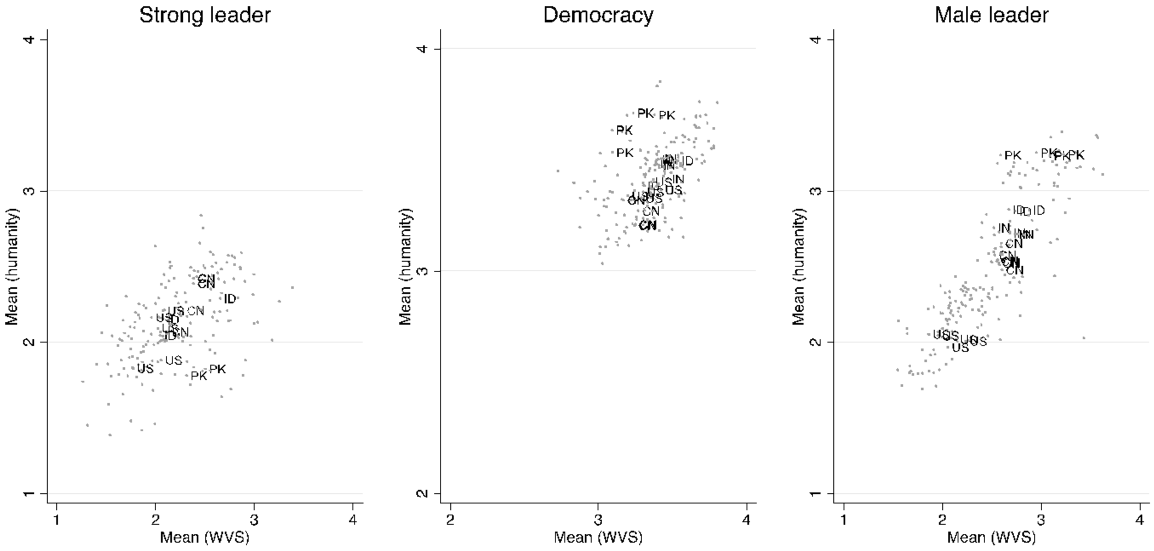

Note: CH=China, IN= India, US=United States of America, ID=Indonesia, and PK=Pakistan

Finally, we also test the accuracy of Step 2, by comparing the estimates of our outcome variables in the humanity dataset to survey data from other sources. To do so, we rely on data from the Global Barometer (2018), an international survey project similar to the WVS but with much more limited coverage (it covers Latin America from 1995; Asia, Africa, and Eurasia from 2001; and Arab countries from 2006). We compare average levels of support for a strong leader and democracy. The question wording is different in the Global Barometer than in the WVS (see Appendix A in the main text), as follows: "There are many ways to govern a country. Would you disapprove or approve of the following alternatives (1 Strongly disapprove, 2 Disapprove, 3 Approve, 4 Strongly approve): Elections and parliaments are abolished so that the president can decide everything, and A democratic political system. Table SM7 shows the mean levels of support across the two data sources for the countries included in both. The

results show that, although the data come from two completely different datasets, average support for strong leaders and democracy are remarkably similar. The country-by-country difference is very small, especially in the case of support for democracy.

Table SM7. Comparing support: Humanity dataset and Global Barometer Surveys

	**Strong leader**	**Democracy**
Humanity dataset	2.15 (0.25)	3.54 (0.10)
Global Barometer	1.96 (0.53)	3.45 (0.19)
Difference between humanity and Global Barometer	0.19 (0.68)	0.10 (0.23)

Note: Entries are averages at the country/year level. Standard deviation is in parentheses. N = 29 (strong leader), 18 (democracy).

We conclude by comparing our estimates from the humanity dataset to raw or “naïve” estimates form the WVS. For these two last estimates, we simply calculate the mean of the outcome variables for each year (WVS raw) and the mean weighted by the population of the country (WVS weighted). This comparison gives us a measure of how much our estimate of global public opinion deviates from or is consistent with imperfectly sampled responses from WVS surveys. Figure SM4 shows that, although the estimates are not radically different, they often differ substantially, especially for earlier years. This is not surprising given that the country coverage in the WVS is less extensive in the 1990s and early 2000s. Our estimate is also less erratic given that it is not sensitive to countries included in any given wave.

Figure SM4. Comparison between humanity dataset and WVS raw estimates

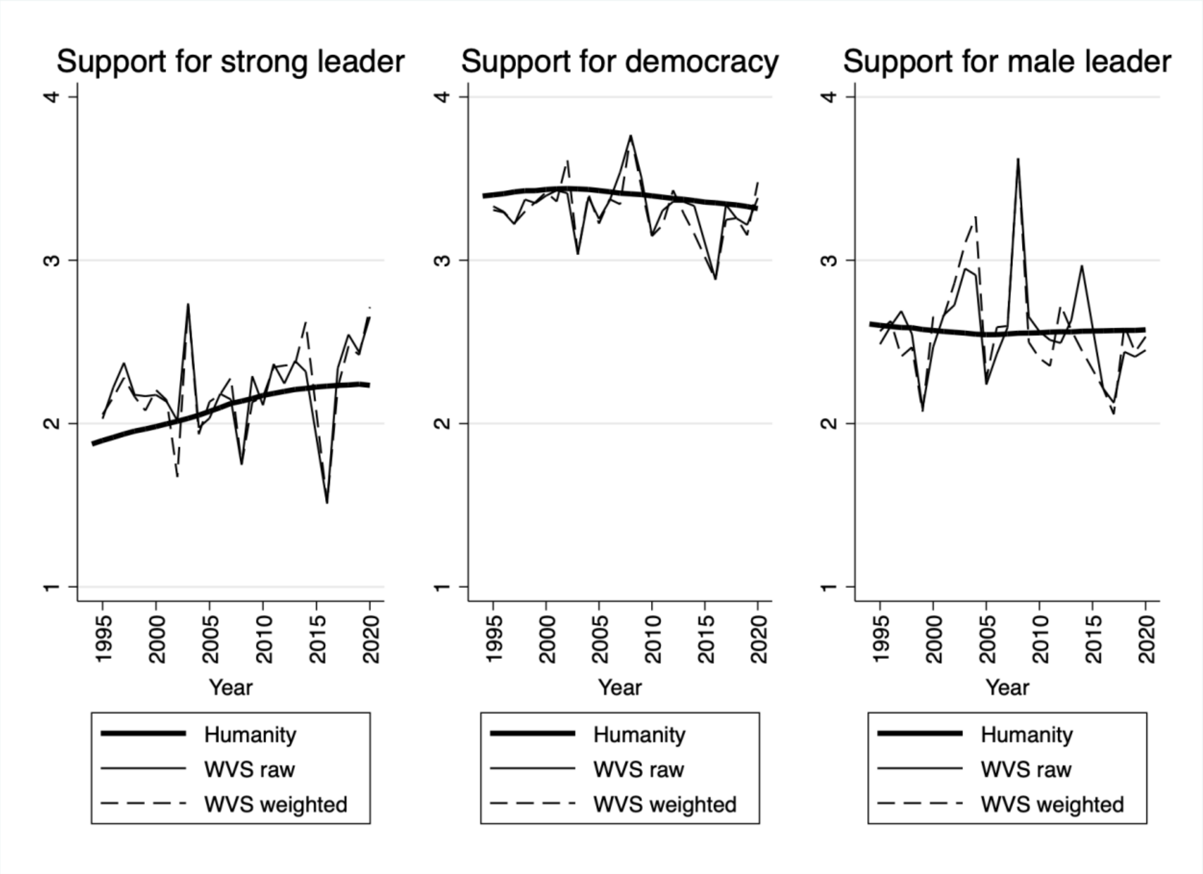

Note: Lines are means.

References

Global Barometer Survey. 2018. Wave 1 & 2 Pooled Datafile, Taipei: Hu Fu Center for East Asia Democratic Studies, NTU [distributor]. https://www.globalbarometer.net/.

King, Gary, and Langche Zeng. 2006. "The Dangers of Extreme Counterfactuals." *Political Analysis* 14 (2): 131-159.
